# Catastrophic Cerebral Infarctions in a Pediatric Patient with Acute Lymphoblastic Leukemia Due to Mucorales Infection

**DOI:** 10.3390/jof11090618

**Published:** 2025-08-25

**Authors:** Alexander M. Aldejohann, Antonio Uribe Munoz, Miriam A. Füller, Grit Walther, Oliver Kurzai, Frieder Schaumburg, Ronald Sträter, Jenny Potratz, Julia Sandkötter, Daniel Ebrahimi-Fakhari, Christian P. Stracke, Laura Beck, Christian Thomas, Andreas H. Groll

**Affiliations:** 1 Institute for Hygiene and Microbiology, University of Würzburg, 97080 Würzburg, Germany; alexander.aldejohann@uni-wuerzburg.de (A.M.A.); antonio.uribe-munoz@uni-wuerzburg.de (A.U.M.); oliver.kurzai@uni-wuerzburg.de (O.K.); 2 Infection Control and Antimicrobial Stewardship Unit, University Hospital Würzburg, 97080 Würzburg, Germany; 3 National Reference Center for Invasive Fungal Infections, Leibniz Institute for Natural Product Research and Infection Biology- Hans Knoell Institute, 07745 Jena, Germany; grit.walther@uni-wuerzburg.de; 4 Department of Pediatric Hematology and Oncology, University Children’s Hospital, 48149 Münster, Germany; miriamantonie.fueller@ukmuenster.de (M.A.F.); ronald.strateter@ukmuenster.de (R.S.); daniel.ebrahimi-fakhari@ukmuenster.de (D.E.-F.); 5 Institute of Medical Microbiology, University Hospital, 48149 Münster, Germany; frieder.schaumburg@ukmuenster.de; 6 Department of General Pediatrics, University Children’s Hospital, 48149 Münster, Germanyjulia.sandkoetter@ukmuenster.de (J.S.); 7 Department of Clinical Radiology, University Hospital, 48149 Münster, Germany; paul.stracke@ukmuenster.de (C.P.S.); laura.beck@ukmuenster.de (L.B.); 8 Institute for Neuropathology, University Hospital, 48149 Münster, Germany; christian.thomas@ukmuenster.de

**Keywords:** mycoses, mucor, children, cancer, infarction

## Abstract

Mucormycosis is a rare invasive fungal disease in pediatric patients with hematological malignancies and is associated with poor outcomes. We present a fulminant and ultimately fatal case of rhino-orbito-cerebral mucormycosis, addressing important issues including clinical signs and symptoms, diagnostic approaches and the challenges of timely diagnosis. The patient was an 11-year old girl undergoing re-induction chemotherapy for Central Nervous System relapse of B-cell precursor acute lymphoblastic leukemia. She presented six days into the second course of chemotherapy in profound neutropenia with aggravating headaches, painful abducens nerve palsy and anisocoria. At first (day −3), no significant radiological or ophthalmological correlations were found, and methyl–prednisolone was started due to suspected vasculitis following ICU admission. After further clinical deterioration, a second MRI scan (day 0) revealed a prolonged occlusion of the left carotid artery, which was successfully stented in a neuroradiological intervention (day +1). However, during the next day the child developed clinical signs indicating severe cerebral dysfunction. An emergency CT scan showed complete infarction of the left hemisphere including a progredient perfusion deficit and beginning brain edema. Based on the unfavorable prognosis, best supportive care was initiated, and the patient deceased on day +2. Pathological and microbiological workup identified thrombotic infarction in all major cerebral arteries. While microscopy was suspicious for mucormycosis, nested PCR from retained blood specimens confirmed the genus *Lichtheimia*. Final NGS on brain tissue led to the identification of *Lichtheimia ramosa.* This case illustrates the rapidity and severity of Mucorales infection. It shows the importance of early clinical suspicion and the need for an aggressive laboratory testing algorithms. The stratification of risk factors and definition of red flags may be a future task fighting these infections.

## 1. Introduction

Mucormycosis is a severe invasive fungal disease (IFD) caused by molds from the order Mucorales with *Rhizopus*, *Mucor,* and *Lichtheimia* being the most frequently observed genera [1,2,3]. In a comprehensive French national survey, mucormycosis accounted for 3% of all IFDs between 2012 and 2018 with a total of 314 cases during the study period [4]. Of these, 13 were reported in children, which suggests that the disease is a rather rare fungal entity in the pediatric population [4].

With up to 40%, most cases of mucormycosis in adults occur in patients with diabetes mellitus, followed by immunocompromised patients treated for hematological malignancies and patients post hematopoietic cell or solid organ transplantation [2]. Important predisposing factors associated with the development of mucormycosis in these patients are corticosteroid use, granulocytopenia, and cancer chemotherapy [2]. Data concerning prevalence and risk factors for pediatric patients are scarce. However—unlike in adults—hematological malignancies, hematopoietic cell transplantation, and solid organ transplantation seem to be the leading underlying conditions [1]. The most common clinical entity in immunocompromised adults and children is pulmonary mucormycosis, followed by other entities such as rhino-orbito-cerebral, cutaneous, and disseminated disease, although the clinical presentation may vary considerably according to the underlying condition [5].

To diagnose mucormycosis, histopathology and culture remain the gold standard in the clinical mycology lab [6]. However, both methods have certain limitations as they are time consuming, labor intensive and often lack sensitivity [6]. To obtain valid diagnostic specimens, invasive procedures are frequently required, which may be difficult to perform given the complex and critically ill patient population [7]. Molecular methods like qPCR or cell-free DNA sequencing are becoming increasingly important, as they may be in the case of blood as diagnostic specimen-less invasive and may provide a faster diagnosis that is often critical for the institution of appropriate therapies [5,8]. Nevertheless, a careful and accurate risk group definition including clinical red flags for vulnerable patient groups is key to taking full advantage of those assays [5].

The treatment of mucormycosis is challenging. Current guidelines recommend high doses of liposomal amphotericin B as first line agent [9]. Newer azoles like isavuconazole or posaconazole are considered first alternatives or option for step down [9]. Surgical interventions, where appropriate, remain an important element in the management of mucormycosis as several large analyses have demonstrated a beneficial impact on survival [9]. Further recommended adjunctive measures include the modification of the underlying deficiency in host defense (i.e., taper of immunosuppressive therapies, particularly corticosteroids in immunosuppressed and administration of hematopoietic growth factors in granulocytopenic patients) [9]. Despite optimal therapy, mucormycosis is a threat to severely immunocompromised patients and—independent of age—carries mortality rates of between 25 and 70% [1,2].

In this case report, we present a fulminant and ultimately fatal case of cerebral mycormycosis in a 11-year-old child treated for recurrent leukemia and address important questions ranging from suspicious clinical signs and symptoms to the challenge of a precise and timely microbiologic diagnosis. Furthermore, the value and the relevance of various diagnostic approaches are critically reviewed.

## 2. Case Presentation

**Presentation and clinical course.** The patient was an 11-year-old girl diagnosed with acute precursor B cell lymphoblastic leukemia (pB-ALL) with Central Nervous System (CNS) involvement on day −468 and treated according to the EsPhALL2017 protocol [10] after confirmation of a BCR/ABL translocation. Treatment included glucocorticosteroids and chemotherapy per protocol, continuous administration of imatinib, and CNS irradiation, and was complicated by pseudomembranous colitis (day −342), a port-catheter related sepsis with *Klebsiella variicola* (day −292), and a prolonged episode of encephalopathy attributed to the therapeutic intrathecal administration of methotrexate (days −202 through −186).

Shortly after the start of maintenance chemotherapy and during scheduled craniospinal irradiation, approximately one year and two months after the initial diagnosis, an isolated CNS relapse was diagnosed (day −27). The patient received intrathecal therapy consisting of cytarabine and prednisolone and was started on a five day course of dexamethasone, followed by systemic re-induction chemotherapy with dexamethasone, vincristine, methotrexate, pegylated asparaginase and a second intrathecal administration of cytarabine and prednisolone according to the F1 re-induction course of the ALL-REZ-BFM 2012 protocol [11] (day −22 through day −18). After completion of chemotherapy, the patient was discharged on day −18 to outpatient care. Antifungal prophylaxis consisted of liposomal amphotericin B 2.5 mg/kg twice weekly due to the potential interactions of the generally recommended mold-active antifungal triazoles with vincristine.

Four days later (day −14), the patient was re-admitted with fever and grade II oropharyngeal mucositis to the Pediatric Oncology ward in a conventional two-bed patient room and started on empirical antibacterial therapy with piperacillin/tazobactam and teicoplanin. After definite defervescence, antileukemic treatment was continued per protocol from day −8 through day −4 with the second re-induction course (F2), consisting of dexamethasone, vincristine, high dose cytarabine, pegylated asparaginase, and a third intrathecal administration of cytarabine and prednisolone. The cerebrospinal fluid (CSF) chemistry and cytology on day −4 were within normal limits and confirmed the absence of leukemic blasts in the CSF. Notably, the patient had been neutropenic (absolute neutrophil count below 500 cells/uL) from day −10 onward and had received a total of 15 days of dexamethasone at a daily dose of 20 mg/m^2^ with the completion of the second re-induction course.

**Figure 1:**
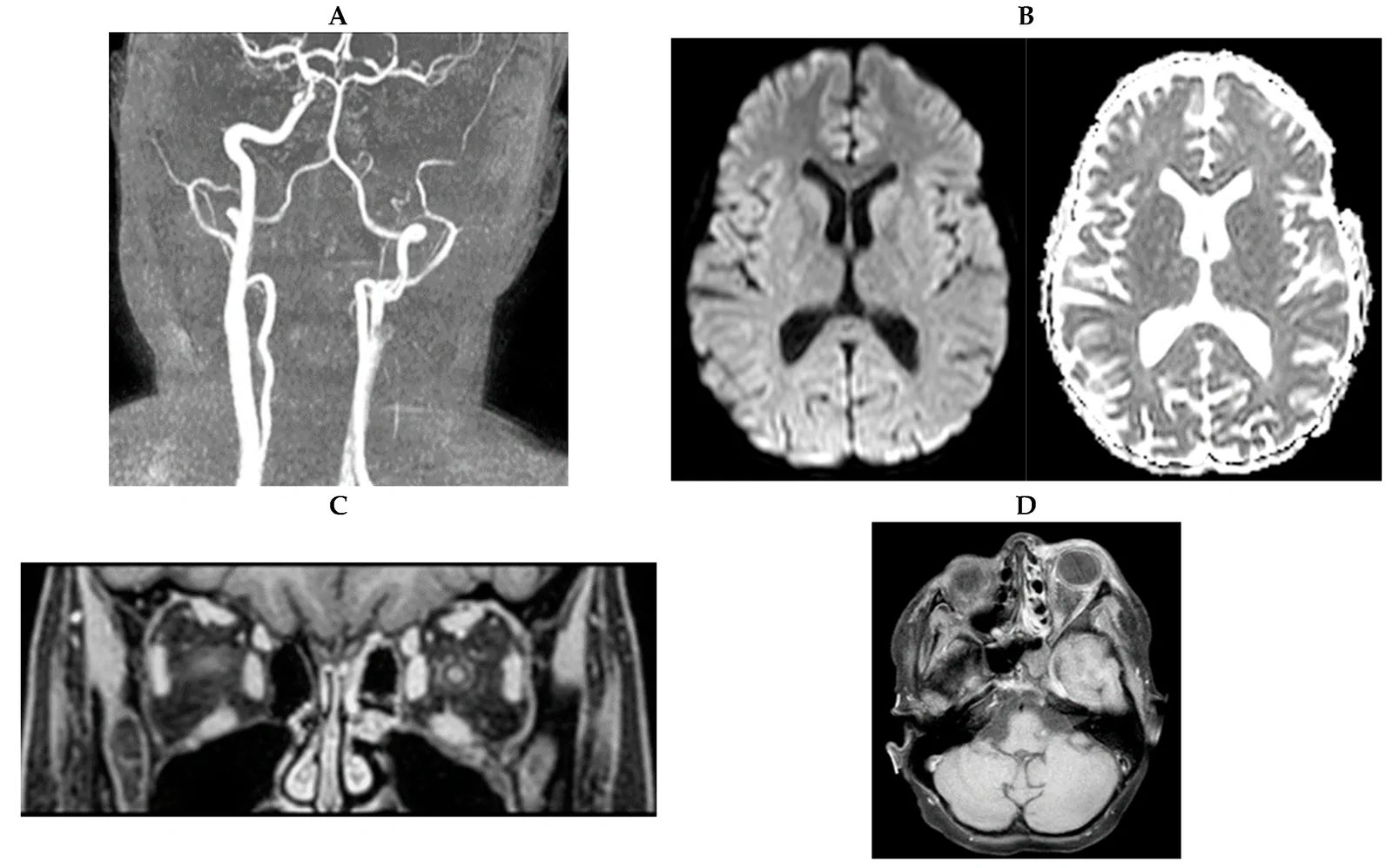
**Magnetic resonance (MR) imaging on day 0.** (**A**), time-of-flight (TOF)-angiography of the cerebral arteries reveals occlusion of the left internal carotid artery. (**B**), diffusion-weighted-imaging (DWI)/ apparent diffusion coefficient (ADC)-Map shows the absence of acute ischemia as explained by sufficient collateralization from the right cerebral arteries. (**C**), after administration of gadolinium, coronar T1w-mDIXON reveals swelling of the surrounding structures of the left optic nerve, and (**D**), swelling and contrast enhancement of the left intraorbital muscles.

During the next two days (days −3 and −2), the patient presented with nausea and progressive headaches with bilateral retrobulbar pain, left abducens nerve palsy and anisocoria. Contrast-enhanced MRI of the head with angiography on day −2 revealed no major findings apart from a discrete swelling of the left optic nerve and a discrete swelling with increased fluid attenuated inversion recovery (FLAIR) signal of the left intraorbital muscles and prominent signs of chemotherapy-induced leukencephalopathy; an ophthalmological consult remained without relevant additional findings. Because of the new onset of arterial hypertension, amlodipine was started. On day −1, there were no changes in the clinical and ophthalmological findings except for a discretely diminished bilateral visual acuity and persistent arterial hypertension that was only controlled by the combination of amlodipine, doxazosine and metoprolol. During the ensuing night, however, deterioration of bilateral visual acuity was noted together with progressive confusion and impairment of vigilance. Based on the primary clinical assumption of a posterior reversible encephalopathy syndrome (PRES), a repeat contrast-enhanced MRI of the head with MR-angiography was prompted in the early morning of the next day (day 0). It revealed no evidence of PRES but a prolonged occlusion of the left internal carotid artery with at that timepoint adequate collateral blood supply and no sign of cerebral ischemia *(*Figure 1A,B). Again, swelling of the left optic nerve and of the left intraorbital muscles was noted (Figure 1B,C).

Physical examination in the morning of day 0 was significant for further deterioration of vigilance, decreased motor strength of the right upper and lower extremities, visible inflammation of the left orbital region, and complete loss of vision of the left eye with unresponsiveness to light. Laboratory evaluation revealed pancytopenia and an elevated C-reactive protein (22.1 mg/dL; upper limit of normal: <0.5) but no relevant abnormalities in clinical chemistry, coagulation, and molecular testing for neurotropic viruses. The patient was started on ceftriaxone and, due to the suggestion of an additional stenosis of the right internal carotid artery by Doppler sonography, on therapeutic enoxaparin and high-dose methylprednisolone (20 mg/kg/day) for possible vasculitis. Following transfer to the intensive care unit for institution of non-invasive ventilation and continuous monitoring, loss of vision also of the right eye and right hemiparesis were noted in the late afternoon. A cranial contrast-enhanced computed tomography scan, however, revealed no new imaging findings apart from a discretely reduced diameter of the cerebral vessels in the area of the left middle cerebral artery, interpreted as possible signs of vasculitis.

**Figure 2:**
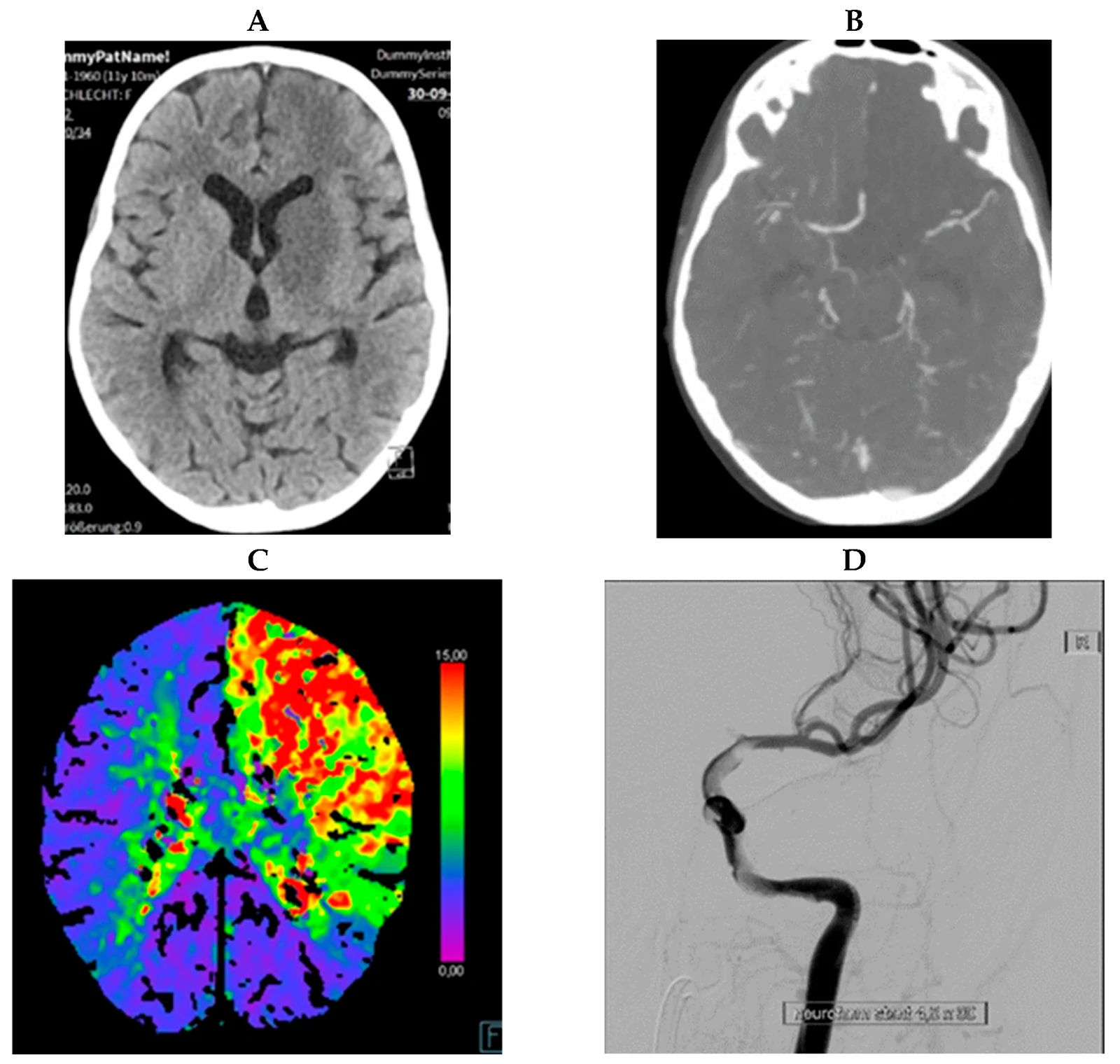
**Computed tomography (CT) and magnetic resonance (MR) imaging on day +1.** (**A**), axial CT imaging showing infarction in the perfusion area of the left anterior (ACA) and middle cerebral arteries (MCA). (**B**), CT angiography showing the extension of the occlusion of left internal carotid artery into the proximal left anterior and middle cerebral arteries. (**C**), time-to-drain (TTD) documents extensive perfusion deficits in the left frontotemporal area. (**D**), postprocedural angiography following interventional thrombectomy and stenting of the left internal carotid artery.

**Figure 3:**
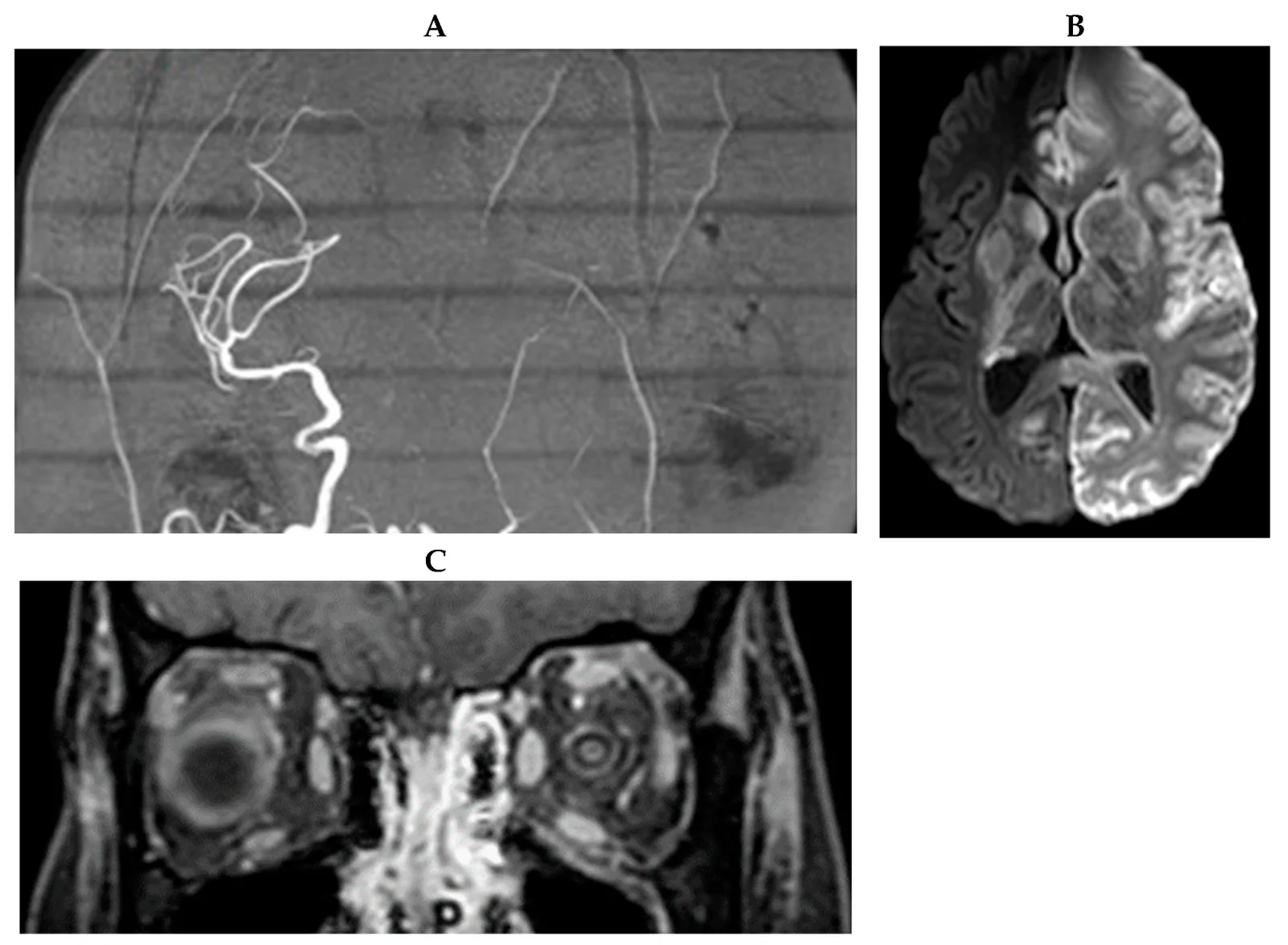
**Angiography and magnetic resonance (MR) imaging on day +2.** (**A**), TOF-angiography reveals perfusion only via the right internal carotid artery and middle cerebral and proximal parts of the basilar artery (BA). (**B**), DWI/ADC-Map showing extensive diffusion restriction of the entire left hemisphere and the right basal ganglia. (**C**), coronar T1w-mDIXON MRI post administration of gadolinum documenting the persistent swelling of the left optic nerve and the left intraorbital eye muscles.

The next morning (day +1), the patient was in coma with dilated pupils unresponsive to light, positive Babinski sign and loss of defensive reflexes. Repeat CT imaging revealed additional complete occlusion of the left proximal anterior cerebral artery (ACA) and partial occlusion of the left middle cerebral artery (MCA) with signs of partial left frontotemporal infarction (Figure 2A–C). In an attempt to rescue as much brain tissue as possible, an interventional thrombectomy of the left ACA and MCA was performed with subsequent stenting of the left ACA and placement of an intracranial Spiegelberg pressure sensor. Postinterventional imaging revealed no evidence of residual perfusion deficits (Figure 2D). In the morning hours of the following day (day +2), autonomous dysregulation was noted. Imaging by transcranial Doppler sonography revealed loss of perfusion of the left ACA and MCA, respectively, and additionally of the right ACA and right posterior cerebral artery (PCA). CT imaging showed a complete infarction of the left hemisphere with a progredient perfusion deficit and beginning brain edema. Subsequent MRI angiography confirmed a nearly complete block of cerebral perfusion, with the only two functional arteries being the right MCA and the right posterior inferior cerebellar artery (PICA) and, at the time, there was suspicion of left intraorbital leukemic infiltration *(*Figure 3A–C).

In view of these detrimental findings, and after parental informed consent, all life-prolonging measures were terminated, and the patient deceased immediately after extubation in respiratory arrest.

**Figure 4:**
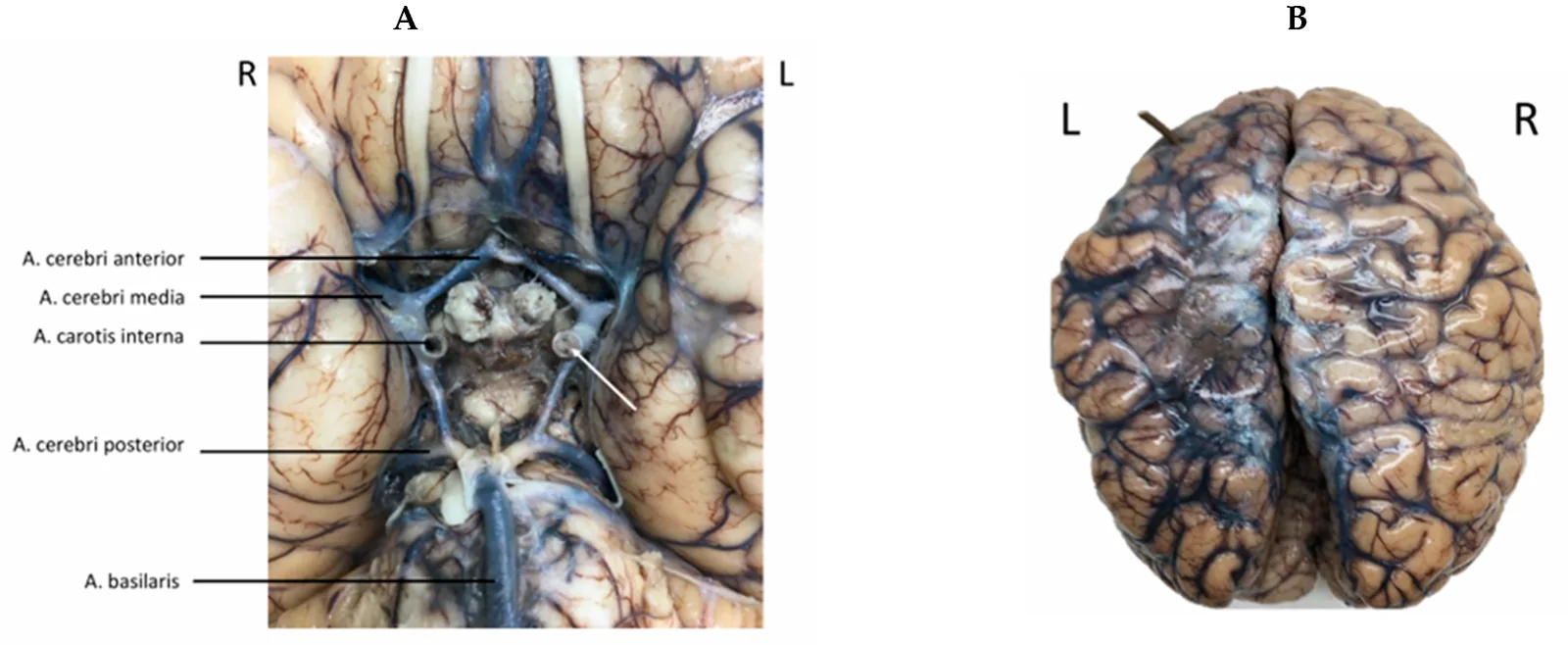
**Macroscopic morphology of the brain at postmortem.** (**A**), thrombotic occlusion of the left internal carotid artery, the left MCA, the right ACA, both PCAs and the basilar artery; (**B**), pronounced diffuse brain edema.

**Figure 5:**
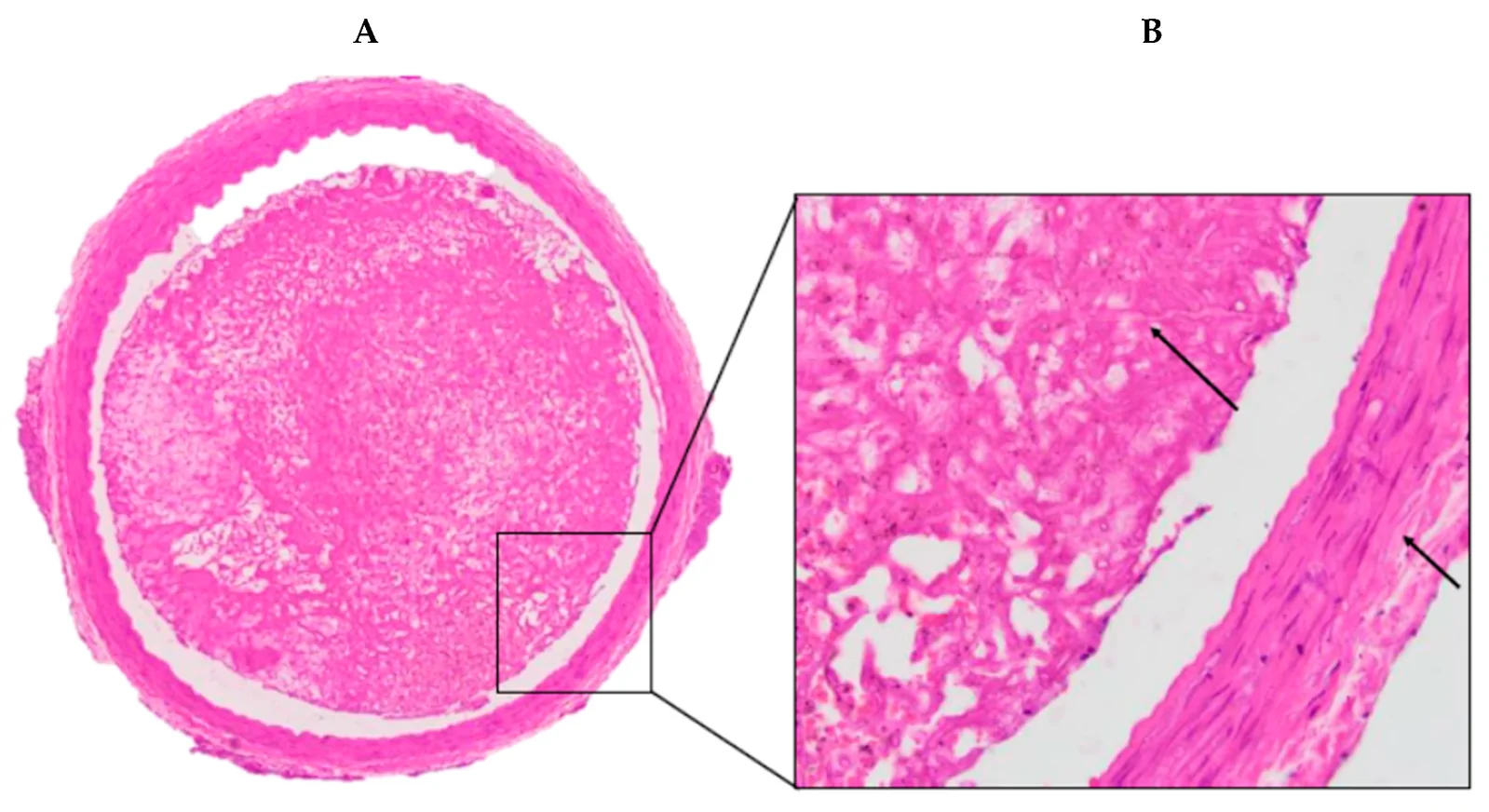
**Microscopic morphology of the Circulus Willisii.** (**A**), intraarterial thrombus of the left internal carotid artery; (**B**), higher magnification reveals irregular ribbon-like twisted hyphae (arrows) within the thrombus (PAS stain, ×10).

**Figure 6:**
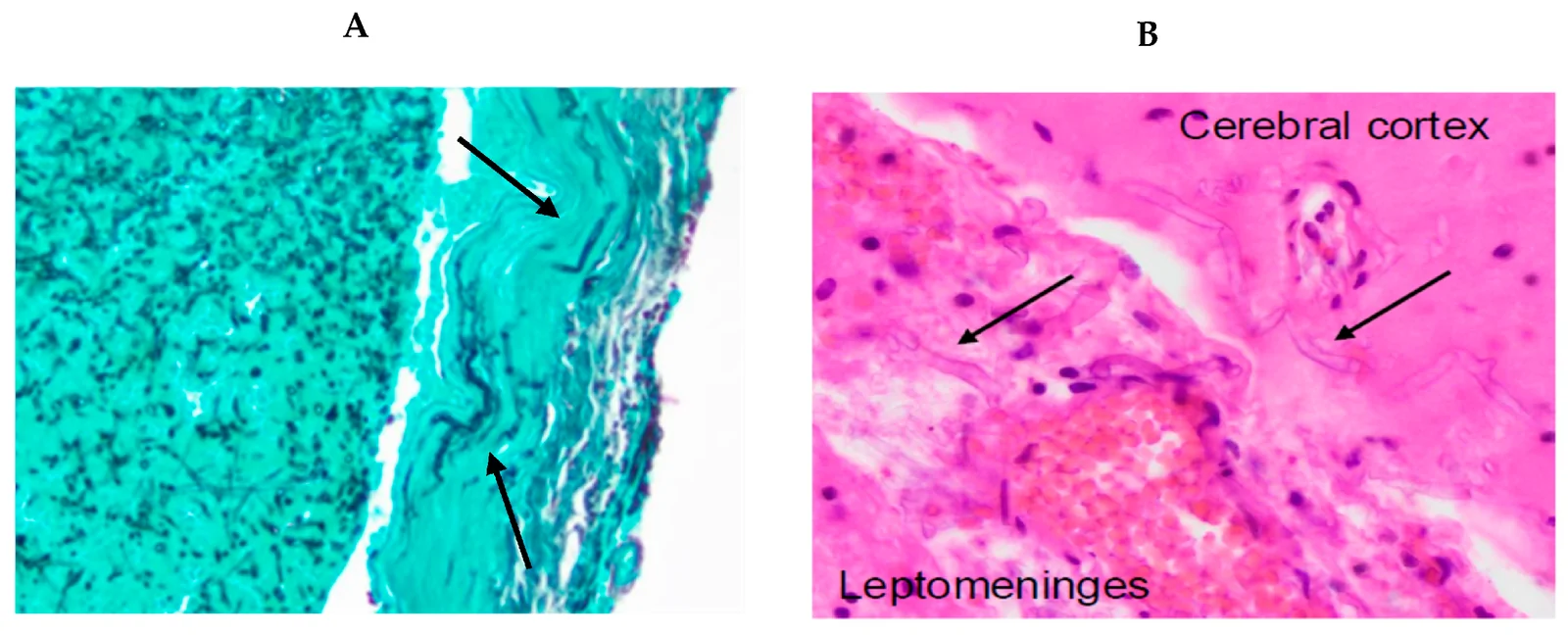
**Microscopic morphology of the Circulus Willisii and the left temporal cortex and surrounding meninges.** (**A**). Invasive hyphal structures (arrows) within arterial wall structures (Grocott stain, ×10); (**B**), hyphal structures (arrows) within necrotic tissue of the left temporal cerebral cortex and the meninges (PAS stain, ×10).

**Histopathological and mycological follow-up**. Due to the child’s sudden deterioration and death without sufficient clinical explanation, a limited postmortem autopsy of the brain was performed following parental consent. Macroscopic examination revealed extensive thrombotic occlusion of the left internal carotid artery, left MCA, right ACA, both PCAs, and the basilar artery, accompanied by marked cerebral edema as the likely cause of death (Figure 4A,B). Histopathological analysis demonstrated multiple infarcts (stage I), predominantly on the left side, and thrombi within major cerebral vessels composed of densely packed, irregular, ribbon-like, and twisted fungal hyphae infiltrating the arterial walls (Figure 5A,B). Similar invasive fungal structures, exhibiting variable diameters (5–25 µm), were also identified within necrotic regions of the optic chiasm, mammillary bodies, left temporal cortex, and adjacent leptomeninges (Figure 6A,B). Based on their variable and wide angle of branching and caliber, the microscopic morphology of the hyphae was suggestive with those observed in mucormycosis.

Genomic DNA was extracted from formalin-fixed paraffin-embedded (FFPE) tissue using a Maxwell Promega device (Maxwell 16 FFPE Plus RSC Kit; Promega, Madison, WI, USA) and subjected to enzymatic fragmentation. Following ligation of universal adapters, random PCR amplification was performed using Twist Unique Dual Index (UDI) primers. The resulting library was sequenced on a MinION Mk1B device (Oxford Nanopore Technologies, Oxford, UK), generating 17,931,197 reads in total. Data analysis was conducted using the ID-Seq pipeline (PMID: 33057676). A total of 2166 reads were assigned to the genus Lichtheimia. Of these, 2093 reads aligned to *Lichtheimia ramosa*, 21 reads were classified as Lichtheimia corymbifera, and the remaining reads were only classifiable at the genus level. The alignments to *L. ramosa* (strain KPH11) in the NCBI NT database yielded an E-value of 10^−78^, indicating highly significant sequence homology. While a small proportion of reads showed classification within the genus Lichtheimia but could not be confidently assigned to a species, no substantial alignment to other species within the order Mucorales was observed.

These results were subsequently confirmed by a nested PCR approach targeting the ITS (internal transcribed spacer) region and the 18S rRNA region using Mucorales specific primers (ZM1 and ZM2) [12,13]. While the analysis of de-paraffinated tissue of the ICA was unsuccessful due to contaminating fungal DNA in the ITS- PCR and no amplicon in the mucorales specific assay; however, the analysis of processed banked serum and whole blood material identified the genus *Lichtheimia* by the amplified ZM1 sequence. ITS-targeting was not evaluable due to low quality unspecific sequences.

During the admission (day −14 to day +2), blood cultures (one pair of aerobic/anaerobic bottles, two pediatric bottles) were taken; all were flagged negative after five days of incubation.

## 3. Discussion

Mucormycosis in children is a rare fungal entity based on case reports and small case series [1,13]. Compared to the epidemiology of adult patients, where some authors report elevated incidence rates, the paucity of data precludes the assessment of epidemiological dynamics in children [1,14,15,16]. However, a more recent study provides valuable insights through the combination of data from two international databases that enabled the analysis of 63 cases of pediatric mucormycosis between 2005 and 2014 [1]. Concordant with the presented case, hematological malignancies and neutropenia were found to be the dominant predisposing factors in children. Furthermore, taken together, the paranasal sinus/sino-orbital (15.8%) and rhino-cerebral regions (7.9%) were the most commonly affected sites of infection; infections of the lungs and the skin and soft tissue were noted in 19% of the cases each. An overall high mortality rate of 33% was reported and the combination of systemic antifungal therapy with surgery was associated with better survival. Interestingly, the mortality rate rose to 66% in children suffering from malignancies and was up to 100% in children not receiving therapy, underlining the importance of early identification, diagnosis and treatment [1].

The clinical diagnosis of mucormycosis is challenging [6]. Symptoms like diplopia with signs of paranasal sinus or orbital inflammation in patients with diabetic ketoacidosis, pleuritic chest pain in patients with immunosuppression or treated for hematological malignancies, or suspected fungal breakthrough infection in patients receiving antifungal prophylaxis without activity against Mucorales, are definite red flags and should promptly lead to further diagnostic investigation [6]. In diabetic adults, algorithms for the early diagnosis of rhino-orbito-cerebral mucormycosis have been proposed. These comprise—among others—cranial nerve palsy, diplopia, sinus pain, proptosis and periorbital swelling [17]. On the contrary, neutropenic children in particular may lack these symptoms and red flags are poorly defined.

In the presented case, severe headaches, palsy of the abducens nerve and anisocoria of the left eye on day −2 were the first symptoms. While imaging was immediately performed, it did not provide unequivocal hints for invasive fungal disease. The discrete swelling of the left optic nerve and of the left intraorbital muscles was interpreted as possible residual of CNS leukemia, and thus had no consequences on antifungal therapy. Although these findings in retrospect have to be interpreted as manifestations of mucormycosis, several other possible differential diagnoses such as myositis, vasculitis, or focal manifestations of CNS leukemia would have been at least as likely. Nevertheless, the appearance of visible inflammation of the left orbital region on day 0 and the concordant cerebral artery infarction might have been a critical flag for invasive mold disease considering the background of the patient’s underlying disease and the history of prolonged steroid administration and profound neutropenia. However, given the fulminant course over the ensuing two days and the persistent immunosuppression, it remains questionable whether the institution of appropriate pre-emptive antifungal therapy would have predictively resulted in a different outcome.

While clinical signs and imaging, as well as suitable biomarkers, provide important information for the potential presence of tissue-invasive fungal disease, the cornerstones of diagnosis rely the detection of the organism by microscopy or histology, microbiological culture and molecular assays from infected tissue [18]. Microscopy and histology allow us to identify the presence of invasive fungal structures and may allow for the tentative differentiation between Aspergillus and Mucorales species, provided that the quality of the sample is good and the examiner is experienced in fungal morphology. As to detection by culture, only 50% of positive histopathologic samples show culture growth, which may be related to the fungal hyphae’s frailty [6]. If, on the other hand, the culture is ultimately positive, Mucorales tend to show prolonged growth behavior of at least 3–7 days, which—as our case demonstrates—might be too late for providing the critical indication for treatment. In this context, molecular assays are rapid add-on diagnostic tools that allow to identify Mucorales at the species level. However, as it occurred in the presented case, the performance may vary depending on the sample (fresh tissue vs. paraffin embedded tissue vs. serum or plasma), sample preparation and a certain risk of contamination [6,19,20]. Again, projected to the case presented, all diagnostic approaches most likely would have come too late to ensure a timely diagnosis and targeted antifungal treatment.

In conclusion, this case illustrates the potentially fulminant nature and severity of Mucorales infection in immunocompromised children and the ultimate lack of a provenly effective antifungal prophylaxis against these organisms. It shows the importance of early clinical suspicion and screening [21] and the need of a well-orchestrated and diverse laboratory testing algorithm to initiate adequate therapy. The further stratification of risk factors and definition of red flags may be a future task to effectively overcome these infections. In the presented case, the possible portal of entry of the infection was the left paranasal sinus with subsequent affection of structures of the left orbit and invasion of the chiasma opticum and vascular structures of the Circulus Willisii with thrombotic occlusion and subsequent infarction of the dependent brain areas.

## Data Availability

The original contributions presented in this study are included in the article. Further inquiries can be directed to the corresponding author.
