# Correction: Aldejohann et al. Catastrophic Cerebral Infarctions in a Pediatric Patient with Acute Lymphoblastic Leukemia Due to Mucorales Infection. *J. Fungi* 2025, *11*, 618

**DOI:** 10.3390/jof11100744

**Published:** 2025-10-17

**Authors:** Alexander M. Aldejohann, Antonio Uribe Munoz, Miriam A. Füller, Grit Walther, Oliver Kurzai, Frieder Schaumburg, Ronald Sträter, Jenny Potratz, Julia Sandkötter, Daniel Ebrahimi-Fakhari, Christian P. Stracke, Laura Beck, Christian Thomas, Andreas H. Groll

**Affiliations:** 1 Institute for Hygiene and Microbiology, University of Würzburg, 97080 Würzburg, Germany; alexander.aldejohann@uni-wuerzburg.de (A.M.A.); antonio.uribe-munoz@uni-wuerzburg.de (A.U.M.); oliver.kurzai@uni-wuerzburg.de (O.K.); 2 Infection Control and Antimicrobial Stewardship Unit, University Hospital Würzburg, 97080 Würzburg, Germany; 3 National Reference Center for Invasive Fungal Infections, Leibniz Institute for Natural Product Research and Infection Biology-Hans Knoell Institute, 07745 Jena, Germany; grit.walther@uni-wuerzburg.de; 4 Department of Pediatric Hematology and Oncology, University Children’s Hospital, 48149 Münster, Germany; miriamantonie.fueller@ukmuenster.de (M.A.F.); ronald.strateter@ukmuenster.de (R.S.); daniel.ebrahimi-fakhari@ukmuenster.de (D.E.-F.); 5 Institute of Medical Microbiology, University Hospital, 48149 Münster, Germany; frieder.schaumburg@ukmuenster.de; 6 Department of General Pediatrics, University Children’s Hospital, 48149 Münster, Germanyjulia.sandkoetter@ukmuenster.de (J.S.); 7 Department of Clinical Radiology, University Hospital, 48149 Münster, Germany; paul.stracke@ukmuenster.de (C.P.S.); laura.beck@ukmuenster.de (L.B.); 8 Institute for Neuropathology, University Hospital, 48149 Münster, Germany; christian.thomas@ukmuenster.de

In the original publication [1], a mistake was made, and the article was published online without the required ethical statements in the back matter. The authors did supply these statements at submission, but they were inadvertently omitted from the final proofs. The newly added part is listed below. The authors have agreed to the correction, and the correction was approved by the Academic Editor.
